# Pathological Society of London

**Published:** 1851-06

**Authors:** 


*Pathological Society of London.*—Mr. Canton exhibited three specimens of *Chronic Rheumatic Arthritis of the Knee-joint.* The first was removed from an elderly woman, who for some time had suffered from the symptoms characterizing this complaint. The outer condyle of the femur presented a series of vertically disposed ridges and furrows alternately arranged. The posterior surface of the patella showed the same peculiarity, so that when the two were in opposition, an inter-locking was the result, but at the same time ginglymoid motion was freely executed. The encrusting cartilage had disappeared from both these surfaces, and porcellaneous material occupied its place. The second specimen was from a man of sixty years of age, and showed the same peculiarities, with the exception of the establishment of surfaces of eburnation to a less degree, though the cartilage had been absorbed to the like extent; the subjacent bone was in part porous, and in part porcellaneous ; a loose cartilage was found in this joint, of an oval form, and situated near the intercondyloid notch. The third articulation was from a middle-aged man, who had long labored under the symptoms of chronic rheumatic arthritis in the knee and hip of the same side. In the latter joint the disease had committed extensive ravages. In the ligamentous tissues, larger and small irregularly shaped osseous growths were to be seen; and around the tibial glenoid cavities similiar deposits had considerably deepened the surface for the femoral condyles. The external semilunar cartilage was wholly removed, so that the femur and tibia were here in contact. Their opposed surfaces were much expanded, and covered by a continuous and uneven stratum of ivory-like deposit. A large number of loose and adherent bodies were in the joint, and varied in consistence from the cartilaginous to the osseous.

Mr. Prescott Hewett exhibited a specimen of *omental sac in a scrotal hernia*, and remarked, that he had great pleasure in being able to bring it before the Society, as it served to confirm the accuracy of the opinion, which he had ventured to express in a paper published some years back, in the *Medico-Chirurgical Transactions*. In speaking of the formation of complete omental sacs in hernia, he had thus decribed the origin of one variety of this disease :—

"An epiplocele takes place, and the portion of omentum which is protruded becomes altered in structure, and its folds are firmly united to each other by the effusion of lymph ; but within the abdominal cavity, in the neighborhood of the ring, the folds into which the omentum has been drawn may not be agglutinated—they will thus leave spaces into which a knuckle of intestine may insinuate itself, pass through the ring, and form for itself a bed in the altered mass of omentum which is in the hernial sac. It may happen that two or three portions of gut may slip into the different spaces left between the folds of the omentum, and subsequently form for themselves separate pouches; several separate sacs, with narrow necks, may be thus found in the omental mass which is in the hernial sac." When that paper was written, Mr. Prescott Hewett had not yet met with any case of this peculiar form of omental sac, neither had he been able to find any published notice of it; the present specimen he found by chance, some two or three years subsequently, in the body of a person who had died of some other disease. In this specimen, the omentum in the hernial sac was of the shape and size of a common pear; it was intimately united throughout to the hernial sac; and the greater portion of it, very much thickened, formed a perfectly solid mass; but close to the external ring, and in the inguinal canal, it was only slightly thickened and drawn into folds, in the centre of which was a well-formed pouch, large enough to lodge a good-sized coil of intestine. One extremity of the pouch reached a little lower than the external ring; the other was in the belly, and about an inch higher up than the internal ring; at this entrance into the pouch, the various folds into which the omentum was drawn had become united to each other by their margins only, and thus formed a complete ring, the circumference of which was very firm in texture, and the size about that of the middle finger; above this ring the omentum was quite healthy in structure. Such a case as this might, it was very evident, become extremely perplexing to the surgeon. In this patient, a convolution of intestine dropping into the pouch thus formed in the omentum, might have become strangulated by the adventitious ring, and the surgeou cutting down upon the hernia in the scrotum would there have formed simply a solid mass of omentum; and had he not proceeded to examine the parts in the neighborhood of the external ring, he would never have discovered the bowel in the omental sac. Even supposing that he had discoveaed this sac, to relieve the strangulation, he would have been obliged to have laid the whole of the inguinal canal freely open, and to have got into the abdomen to divide the neck of the sac, which was an inch higher up than the internal ring, and which might have been the sole cause of the stricture.

